# Coccidioidal meningitis and neurosyphilis co-infection in a non-HIV patient

**DOI:** 10.1016/j.mmcr.2021.08.002

**Published:** 2021-08-09

**Authors:** Jani M. Kim, Geetha Sivasubramanian

**Affiliations:** University of California San Francisco, Fresno Medical Education Program, 155 N Fresno St., Fresno, CA, 93701, United States

**Keywords:** Coccidioidomycosis, Neurosyphilis, Meningitis

## Abstract

*Coccidioides* sp. and *Treponema pallidum* can both cause infections of the central nervous system if untreated. We describe a case of an immunocompetent patient living in an endemic region for *Coccidioides* who presented with headaches and diplopia and was found to have co-infection of coccidioidal meningitis and neurosyphilis. We highlight the importance of evaluation for CNS co-infections as they may be underdiagnosed, especially in endemic areas for coccidioidomycosis.

## 1 Introduction

*Coccidioides immitis* and *Coccidioides posadasii* are dimorphic fungi endemic in central and southern California, the low deserts of Arizona, southeastern New Mexico, western Texas, and several other areas of the southwestern United States [1]. Central nervous system (CNS) infection due to coccidioidomycosis is uncommon but has high mortality if untreated. Patients generally present with progressive headaches, diplopia, and altered mental status [2].

Syphilis is caused by the spirochete bacteria, *Treponema pallidum.* Neurosyphilis is the form that involves the brain or spinal cord [3]. Patients with neurosyphilis present with headaches, seizures, ataxia, as well as psychiatric symptoms including personality changes [4].

There have been case reports of co-infections with neurosyphilis and other fungal infections such as cryptococcosis [5]. Coccidioidomycosis has also been reported as a co-infection in patients with histoplasmosis, tuberculosis, and influenza in the setting of uncontrolled HIV infection [6]. In this article, we present a case of concomitant infection with neurosyphilis as indicated with positive CSF VDRL and *Coccidioides immitis* as documented by positive CSF immunodiffusion and complement fixation.

## 2 Case presentation

A 33-year-old female living in Fresno, California presented to the hospital with progressively worsening diplopia and headache for 5 days. Chart review showed that she had been diagnosed with coccidioidal meningitis three years ago when she presented with similar headaches and reduced visual acuity. Computed tomography (CT) of the head at the time showed hydrocephalus. CSF opening pressure was 52 cm H2O. Coccidioides complement fixation titer of the CSF was positive at 1:16. She was started on oral fluconazole 1000 mg daily for adequate CNS penetration and a ventriculoperitoneal shunt was placed at that time. She was eventually discharged home but lost to follow-up.

**Fig. 1:**
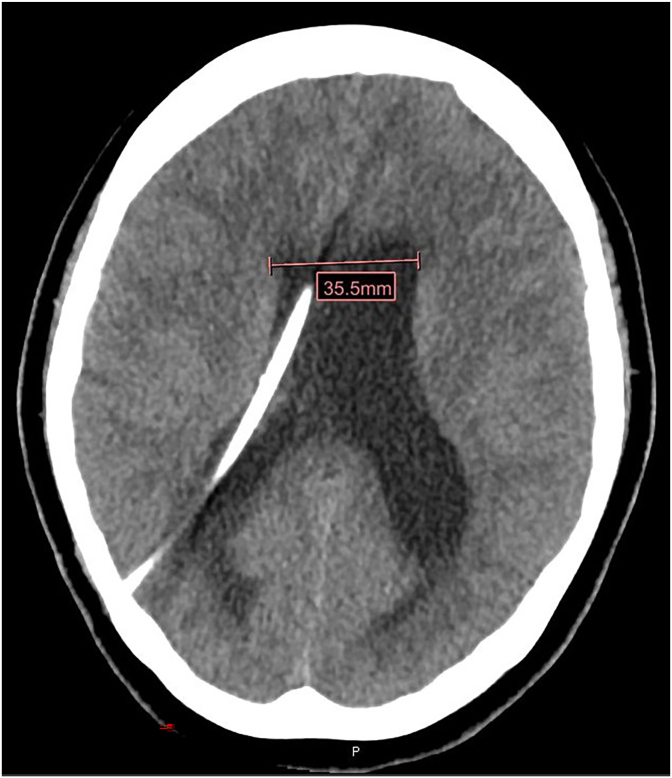
Computed tomography of the head on Day 0 shows mild to moderate hydrocephalus with the presence of a right posterior parietal VP shunt tube that traverses the right lateral ventricle.

In the Emergency Department on Day 0, her vital signs were stable within normal range. Physical exam was benign, other than oblique diplopia. CT of the head showed hydrocephalus and a right posterior parietal ventriculoperitoneal shunt tube (Fig. 1). Lumbar puncture was performed on Day 1. Opening pressure was 17 cm H2O. CSF analysis revealed leukocytes of 51/uL with 69% lymphocyte predominance, glucose 23 mg/dl, protein 324 mg/dl. CSF studies showed positive *Coccidioides* complement fixation at 1:32 and VDRL 1:32. Fungal culture of CSF was negative. RPR titer was 1:32. Upon further investigation, the patient had been diagnosed with syphilis about 2 years ago when she presented to an Emergency Room with vaginal pain and swelling. RPR at that time was positive at 1:16, but the patient had already left the Emergency Department and did not receive any treatment. The Department of Public Health also confirmed that she had never received appropriate treatment for syphilis.

**Fig. 2:**
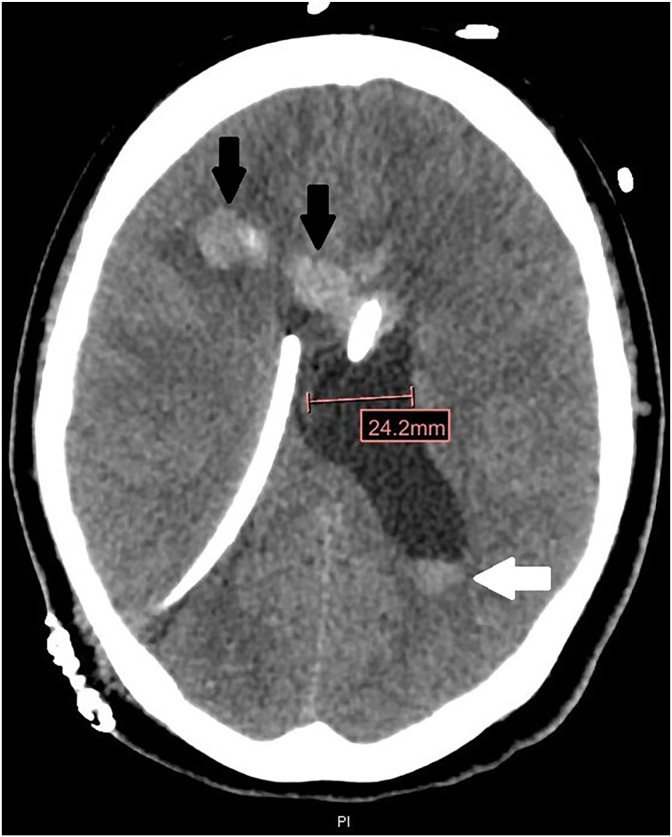
Computed tomography of the head on Day 22 shows dilated left lateral ventricle with parenchymal hemorrhage in the right frontal lobe (black arrows) and intraventricular hemorrhage (white arrow) despite ventriculostomy tubes.

She was started on Fluconazole 1000 mg daily to treat CNS infection with *Coccidioides* as well as Penicillin G 4 million units IV every 4 hours for 14 days to treat neurosyphilis. Unfortunately, her mental status continued to decline requiring intubation for airway protection. On Day 20, she underwent external ventricular drain (EVD) placement due to worsening hydrocephalus on repeat imaging. Subsequent imaging on Day 22 showed worsening ventriculomegaly with bifrontal hemorrhage despite EVD placement (Fig. 2). Due to lack of neurological improvement and poor prognosis, her family elected to transition to comfort-focused care, and she was discharged on a hospice on Day 30.

## 3 Discussion

Coccidioidomycosis, also known as Valley Fever, occurs through inhalation of spores of *Coccidioides* species in endemic areas such as Central California. Although most infections are self-limited, patients can develop severe disseminated disease as coccidioidal spherules or endospores migrate to other areas of the body such as skin, bone and meninges [7]. In patients who present with persistent headache, declining mental status, vision changes with positive serum coccidioidal antibody tests, analysis of CSF must be performed to establish the diagnosis. CSF findings for coccidioidal meningitis include pleocytosis with lymphocytic predominance, elevated protein, and high opening pressure. Other CSF studies like complement-fixation (CF) coccidioidal antibody test which detects the presence of IgG antibody, real-time polymerase chain reaction (RT-PCR), and fungal culture should be performed [2]. The most common complication and mortality factor of CNS coccidioidal infection is hydrocephalus which usually does not resolve with antifungal therapy alone and almost always requires mechanical shunting [8].

Syphilis is caused by the spirochete bacteria, *Treponema pallidum*, which can disseminate to the CNS if untreated. The incidence of syphilis is rising in the Central Valley since 2000 [9]. Presentation of neurosyphilis ranges from asymptomatic to meningovascular syphilis causing hemiplegia, aphasia to parenchymatous syphilis which causes paralysis and tabes dorsalis [3]. Rarely, space-occupying lesions called gumma can occur in the brain or spinal cord although this usually is found in HIV-infected patients [10]. In patients with positive serological tests for syphilis with neurological symptoms, CSF examination is recommended [11]. CSF analysis for neurosyphilis includes elevated WBC with lymphocytic predominance, elevated protein, and reactive CSF-VDRL, although non-reactive CSF-VDRL does not exclude neurosyphilis diagnosis given its variable sensitivity (30–70%). Treatment of neurosyphilis depends on reaching sustained levels of penicillin in the CSF, requiring frequent intravenous dosing. If serologic markers are not decreasing at least four-fold after treatment, repeat CSF analysis should be done [12].

Based on thorough literature review, this appears to be the first case of documented co-infection of coccidioidal meningitis and neurosyphilis. Both infections can cause severe brain damage and neurologic disability if not treated. Therefore, it is critical for timely diagnosis and appropriate management in order to improve patient outcomes [2,3] Although the clinical significance of co-infection of coccidoidal meningitis and neurosyphilis is unclear, it is important to recognize the possibility of dual infections in order to initiate appropriate treatment. Further studies are needed to explore the effect on clinical outcomes of CNS co-infections, especially in endemic areas for coccidioidomycosis where neurosyphilis may be under recognized.

## 4 Conclusion

Coccidioidal meningitis and neurosyphilis are both infections of the CNS that can cause high morbidity and mortality if not treated. Coccidioidomycosis should be in the differential diagnosis in patients with CNS disease in endemic areas. However, CNS co-infections such as neurosyphilis should also be explored in order to provide appropriate treatment in a timely manner.

## Declaration of competing interest

There are none.
